# The Antioxidant Properties of Selenium and Vitamin E; Their Role in Periparturient Dairy Cattle Health Regulation

**DOI:** 10.3390/antiox10101555

**Published:** 2021-09-29

**Authors:** Jianxin Xiao, Muhammad Zahoor Khan, Yulin Ma, Gibson Maswayi Alugongo, Jiaying Ma, Tianyu Chen, Adnan Khan, Zhijun Cao

**Affiliations:** 1 Beijing Engineering Technology Research Center of Raw Milk Quality and Safety Control, State Key Laboratory of Animal Nutrition, College of Animal Science and Technology, China Agricultural University, Beijing 100193, China; xiaojianxin-dairy@cau.edu.cn (J.X.); zahoorcau@cau.edu.cn (M.Z.K.); bs20193040395@cau.edu.cn (Y.M.); bh2020270153@cau.edu.cn (G.M.A.); majiaying@cau.edu.cn (J.M.); chentianyu@cau.edu.cn (T.C.); 2 Faculty of Veterinary and Animal Sciences, The University of Agriculture, Dera Ismail Khan 29050, Pakistan; 3 Shenzhen Branch, Guangdong Laboratory for Lingnan Modern Agriculture, Genome Analysis Laboratory of the Ministry of Agriculture, Agricultural Genomics Institute at Shenzhen, Chinese Academy of Agricultural Sciences, Shenzhen 518000, China; dr.adnan93@cau.edu.cn

**Keywords:** periparturient period, dairy cattle, oxidative stress, antioxidants, selenium, vitamin E

## Abstract

Dairy cattle experience health risks during the periparturient period. The continuous overproduction of reactive oxygen species (ROS) during the transition from late gestation to peak lactation leads to the development of oxidative stress. Oxidative stress is usually considered the main contributor to several diseases such as retained placenta, fatty liver, ketosis, mastitis and metritis in periparturient dairy cattle. The oxidative stress is generally balanced by the naturally available antioxidant system in the body of dairy cattle. However, in some special conditions, such as the peripariparturient period, the natural antioxidant system of a body is not able to balance the ROS production. To cope with this situation, the antioxidants are supplied to the dairy cattle from external sources. Natural antioxidants such as selenium and vitamin E have been found to restore normal health by minimizing the harmful effects of excessive ROS production. The deficiencies of Se and vitamin E have been reported to be associated with various diseases in periparturient dairy cattle. Thus in the current review, we highlight the new insights into the Se and vitamin E supplementation as antioxidant agents in the health regulation of periparturient dairy cattle.

## 1. Introduction

The antioxidant system’s capacity to neutralize and remove reactive oxygen species (ROS) created during metabolic activities is normally sufficient under normal physiological conditions. It has been documented that metabolic changes during pregnancy and calving may raise ROS production beyond the required threshold [1]. Oxidative stress developed when the imbalance between ROS production and the availability of antioxidant molecules occurred, which had proven cattle to various infections [2,3]. The high production of ROS other than the normal level causes lipid peroxidation results in oxidative stress, tissue damage and altering the level of reduced glutathione (GSH), which is involved in glutathione metabolism [4,5]. Damage to the structure and function of cellular macromolecules (lipids, proteins, and nucleic acids) occurs when the pro/antioxidant balance is disrupted, resulting in oxidative stress and a preponderance of oxidation over reduction processes lead to metabolic disorders and diseases in dairy cows [6]. Thus maintaining redox homeostasis in dairy cows is crucial during the periparturient and peak lactation phases [7,8,9].

The oxidative stress around parturition may contribute to immune and inflammatory abnormal function and thus increase the chances of metabolic and infectious diseases [10,11]. The balance between oxidants and antioxidants is necessary during the milking period to improve dairy cows’ efficiency [12]. During the periparturient period, oxidative stress is considered one of the key factors associated with susceptibility to infections such as retained fetal membranes, metritis, mammary edema, mastitis and retained fetal membranes [2,13], which is supported by several studies [14,15,16]. Harrison et al. reported that oxidative stress before parturition is probably the main contributing factor for metabolic and reproductive disorders in the milking period in dairy cattle [17]. Consistently Kizil et al. documented that the acceleration of peroxidation reactions and weakened antioxidant system in cows were associated with metritis [18].

The supplementation of vitamins and minerals has been documented to have a positive impact on the enhancement of the antioxidant status and immune responses in perinatal calves [19]. The multicomponent antioxidative system neutralized the generated ROS, including selenium-dependent glutathione as one of its primary components (GSH-Px; EC 1.11.1.9). The catalytic center of the enzyme contains selenium in the form of selenocysteine, which catalyzes the reduction of hydrogen peroxide and lipid peroxides when combined with GSH [20]. Consistently, the Se and vitamin E have been widely studied for their antioxidative properties and immune regulation and consequent role in cattle health [21,22,23]. Similarly, other studies also documented the positive impact of Se and vitamin E supplementation on the antioxidant and health status improvement of dairy cattle [24,25]. Consistently, it has been documented that Se and vitamin E administration significantly enhanced the growth rate of calves by reducing the perinatal oxidative stress [26].

The periparturient phase in high milking cows requires maximum antioxidant system efficiency, while deficiency of nutrients like selenium and vitamin E expose dairy cattle to placental retention and mastitis [27]. When the milk yield of cow is high, the antioxidant system’s lowering capability of oxidative stress may be insufficient, which is especially obvious in selenium insufficiency. The availability of selenium in the body is the primary determinant of glutathione peroxidase and other selenoproteins involved in various metabolic processes [28,29,30]. Beside, the deficiency of Se and vitamin E also compromised the immune system of periparturient cows. Vitamin E and Se share common biological activities have shown antioxidant properties and deficiencies of these nutrients may lead to neutrophil activity reduction as well. Thus, the deficiencies of either one or both may affect the antioxidant activity of the body which is not sufficient to protect the neutrophil from the destructive action of toxic oxygen molecules in mammary glands [31,32]. The sufficient supply of vitamin E and Se maintained the optimum level of antioxidant activity which is associated with the rapid neutrophil influx into milk during intramammary infection caused by microorganisms [33,34]. Thus, we designed the current review to highlight the consequences of oxidative stress in periparturient dairy cattle and the role of Se and vitamin E supplementation as antioxidant agents in animal health regulation.

## 2. Factors That Predispose Periparturient Dairy Cattle to Oxidative Stress

**Figure 1:**
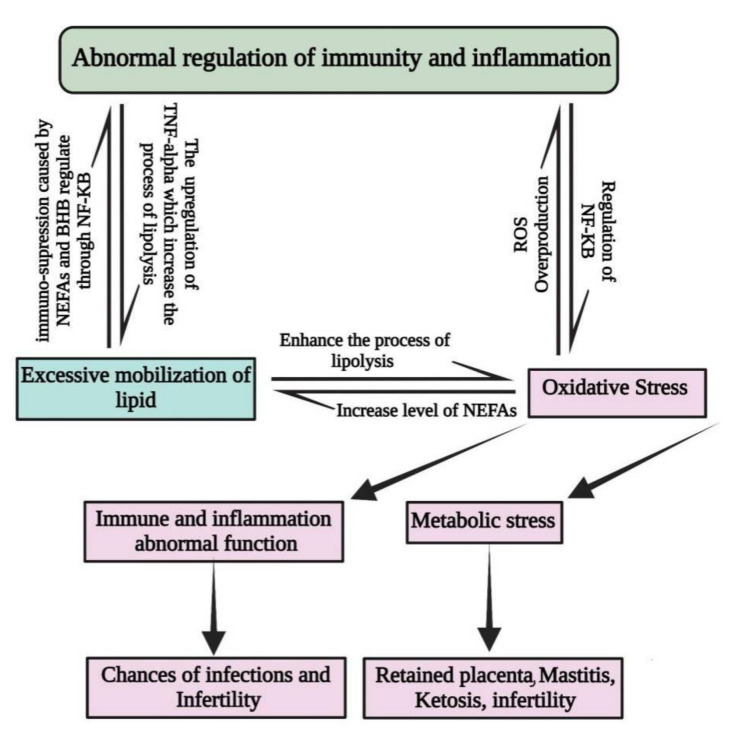
The interconnection among oxidative stress, excessive mobilization of lipid and abnormal regulation of immunity and inflammation; the oxidative stress causes dysfunction of immunity and inflammation by using nuclear factor kappa-B (NF-kB) signaling. The abnormal regulations of inflammation enhance the tumor necrosis factor-alpha production in non-phagocytic cells, resulting in over-production of OS and excessive lipolysis.

During the periparturient period, dairy cattle experience several physiological changes [35]. Metabolic stress is one of the key issues developed due to these physiological changes, which are associated with excessive lipid mobilization followed by oxidative stress and inflammatory and immune dysfunction [36,37]. These three processes (excessive mobilization of lipid, oxidative stress, inflammatory and immune dysfunction) are interconnected, which may contribute to the diseases during the periparturient period in dairy cattle [38,39,40,41]. The interrelation of oxidative stress, excessive lipid mobilization and inflammatory and immune dysfunction are summarized in Figure 1.

The dry matter intake in periparturient dairy cattle decreases and the energy and the calcium requirements increase for lactation [42]. In such condition, more oxygen is utilized by dairy cattle for cellular respiration to fulfill the demand of energy for the onset of lactation, which may lead to a negative energy balance [43]. To fulfill the requirement of energy, dairy cattle utilize body reserves especially in adipose tissues [39]. The increase in the ROS generation and reactive nitrogen species (RNS) may take place in response to excessive lipid mobilization [44,45]. The high intake of crude protein (CP) near parturition in dairy cattle may lead to increased urea level, which is associated with the development of nitrosative stress status, which negatively impacts animal health [44].

The body condition score (BCS) is another important factor determining cows’ susceptibility to OS during the periparturient period. It has been documented that cows with higher body condition scores > 3.5/5 were more prone to OS and metabolic stress at transition time [14]. The BCS is characterized by a breakdown of fat and protein followed by catabolic pathways associated with energy production from lipids and amino acids during the transition period in cattle. Furthermore, it has been shown that lipid peroxidation is considered a key factor for OS [46]. The excessive loss in BCS is also a critical factor associated with excessive production of OS and non-esterified fatty acids (NEFAs) in periparturient dairy cattle [47,48].

The increased level of NEFAs and beta-hydroxybutyrate (BHB) due to lipid mobilization may lead to oxidative stress and dysregulation of the immune system in periparturient dairy cattle [39,49]. A low level of glucose around parturition is also a critical factor that causes less effective pathogen-killing oxidative burst from polymorphonuclear neutrophils [50] which impaired the host defense. The inflammation around peripartal helps to facilitate the process of parturition and homeorhetic adaptations to the onset of lactation [51,52]; however the abnormal regulation of inflammation caused by OS around parturition was documented to be associated with metabolic and infectious diseases [39]. From the above discussion we concluded that nutritional management might be one of the effective ways to enhance the antioxidant capacity of animal and protect them from oxidative stress.

## 3. Antioxidant Properties of Selenium and Their Role in Dairy Cattle Health

For animals, there are two important sources of Se: (1) selenoamino acids naturally derived from plants, such as selenomethionine and selenocysteine; and (2) inorganic Se, such as selenate or selenite [53]. Even if an animal’s physiological requirement for Se is modest, the anti-oxidant system is weakened if it is not satisfied, resulting in negative repercussions for animal health [54]. Selenium is an essential trace element having antioxidants and immunomodulating properties [55,56,57].

Dietary selenium (Se) can be supplemented from organic or inorganic sources and this may affect Se metabolism and functional outcome such as antioxidative status and immune functions in dairy cows [53]. Glutathione peroxidase (GPx) is a selenium-containing antioxidant enzyme that plays an important role in the antioxidant defense of the body [58]. The selenium status is usually determined by measuring the level GPx in blood [59]. Furthermore, selenium supplementation caused an undefined antibacterial activity in milk lactoserum [60], but the mechanism of this antibacterial activity is unknown; however, increased glutathione peroxidase (GSH-Px) activity will decrease pathogenic microorganisms’ development rate in whey. Selenium is a powerful antioxidant that is integrated into selenate in the form of GSH-Px [61]. GSH-Px is a selenoprotein that plays a role in the antioxidative defense process in mammals and cells. It has the ability to eliminate lipid-damaging peroxides and protect immune cells from oxidative stress [57]. Recently published findings showed that supplementation of 0.30 mg Se/kg of DM as Nano-Se for 30 days significantly increased the level of GSH-Px activity. In addition, the mRNA expression of genes (glutathione peroxidase 1, 2 and 4; thioredoxin reductase 2 and 3; and selenoproteins W, T, K and F) were upregulated in response to Nano-Se supplementation in mammary glands of dairy cattle [62].

Se regulates several important antioxidant genes such as TOAX, GPX, CAT, SOD, and GSH to relieve oxidative stress [63]. The SOD gene causes the reduction of oxidative stress through the quenching of the superoxide radical and transferring it into less toxic hydrogen peroxide followed by breaks down into water and oxygen to prevent DNA damage using enzyme catalase (CAT) [64,65,66]. The different types of selenoproteins and their antioxidant properties are summarized in Table 1.

As a food component, selenium is an exceptional agent of protection from atherosclerosis, coronary ischemic disease and cancer. Due to the antioxidant properties, selenium is considered a serious factor of biological and antioxidant protection of vascular endothelium, low-density lipoproteins, DNA protection and chromosomes [77].

### 3.1. Selenium Role in Cattle Health

Oxidative stress is the major contributor to various diseases in dairy cattle, including metabolic and inflammatory problems (mastitis, metritis, ketosis etc.,) [36,78,79,80], and parasitic infections [81,82]. It has been reported that oxidative stress is also associated with reproductive diseases in cattle [83,84,85]. Furthermore, Diyabalanage et al. reported that an adequate supply of Se is necessary for cattle because it can pass through the placental barrier during pregnancy which is essential for intrauterine and calf development [59]. Consistently, a study reported that the sufficient supply of Se could enhance the antioxidative status and consequent improvement in dairy cattle health [86].

#### 3.1.1. Selenium Role in Mastitis Control

High-producing periparturient dairy cows experienced more incidence of mastitis because of oxidative stress which causes the changes in the expression of genes associated with proinflammatory factors [87]. Furthermore, Miranda et al. [88] reported that low levels of Se and glutathione peroxidase activity increase oxidative stress in the mammary gland, which is linked to a reduction in the number of mammary epithelial cells. However, the balance level of Se supplementation decreases the concentration of hydrogen peroxide in mammary epithelial cells [88]. Thus by reducing the level of hydrogen peroxide in mammary epithelial cells, the oxidative status could be relieved which results in reducing the apoptotic cells.

In a recent study GSH-Px activity in whole blood and somatic cell count (SCC) in canned milk have been found to be negatively correlated with each other [89]. The increase in GSH-Px activity in blood after selenium supplementation was linked to a reduction in the frequency of subclinical mastitis in dairy cattle [12]. Consistently, previous studies show that Se supplementation improves antioxidant status; enhances the plasma glutathione peroxidase (GSH-Px) activity, decreases the malondialdehyde (MDA) level in plasma, and decreases SCC in milk [90,91,92,93,94]. The increased level of MDA is an indication of oxidative stress.

Twenty-five selenoproteins have been discovered in animals, and at least 12 of them have a broad immunological and antioxidant role, indicating that they could be useful in dairy cattle udder health and the prevention of subclinical mastitis. The sensitivity of cows’ mammary glands to bacteria may be linked to their selenium levels [89]. According to recent studies, the incidence of mammary gland infection in dairy cows is dramatically reduced after eight weeks of selenium supplementation at a dietary level of 0.2 mg/kg [95]. In general, selenium deficiency suppresses the immune system, whereas supplementing with low amounts of selenium may improve and/or restore immunologic capabilities. In a study, Hemingway found that 14 out of 36 cows receiving antibiotic therapy during the dry time developed mastitis, but only four cows out of 36 receiving 4 mg Se during dry milking had mastitis [96]. A study has documented that Se enhanced the immunity of the mammary gland against infection and subsequent control of mastitis [60]. In addition, they showed that the mammary gland’s innate and adaptive immune action is improved through cellular and humoral activities in response to Se supplementation.

It has been reported in a recent study that supplementation of Se for 90 days in mice decreased the IL-1β, TNF-α, pyrin domain-containing protein 3 (NLRP) and caspase-1 expression level in *Staphylococcus aureus*-infected mice [97]. Moreover, they documented that Se treatment also causes the inhibition of the NF-κB/MAPK pathway by suppressing NALP3 and attenuate the mastitis caused by *S. aureus* in mice [98,99,100,101,102]. The Se also inhibited the expression of TLR2, myeloid differentiation factor-88 (Myd88), NLRP3, Caspase-recruitment domain (ASC), and Caspase-1 caused by *S. aureus* in mice RAW 264.7 macrophages followed by suppression of NF-κB and MAPK signaling pathways [103,104]. Besides, Se also regulates the LR2-related pathways in the mouse mammary gland followed by *S. aureus* infection to suppress the inflammatory and control mastitis. Injectable Na-selenite as Se also decreased the level of somatic cell count in milk and enhanced dairy cattle’s milk production [105].

#### 3.1.2. Effect of Selenium on Reproduction of Animals

It has been reported that oxidative stress severely reduces sperm function while antioxidants such as Se can correct the male infertility factors [106,107]. Besides, the decreased level of selenium is also associated with infertility, anestrous and retained placenta in dairy cattle [108]. The deficiency of selenium is associated with abortions [109,110] and stillbirth [111]. The possible reason for abortion is the insufficient progesterone concentration to maintain the pregnancy. The supplementation of Se has been associated with the improved concentration of progesterone and promotes its postpartum production [110]. Moreover, the incidence of metritis and ovarian cysts [97] and the incidence of retained placenta were decreased in response to Se administration [112]. In addition, the Se also regulates the expression of GPx1 in granulosa cells which has a role of antioxidant during ovarian follicular development [113].

**Figure 2:**
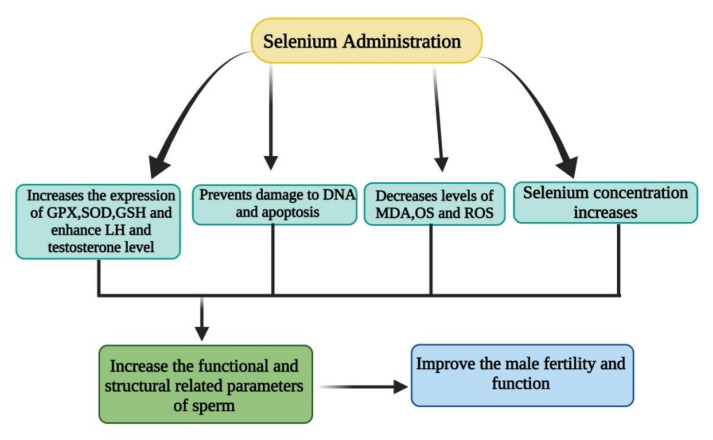
The Se supplementation enhanced the expression of TOAC, GPX, SOD, testosterone and LH; decreased the level of ROS, OS, MDA and consequent DNA damage. The sperm functional and structural parameters are increase and consequent male fertility and reproductive function.

It has been reported that the integrity of the sperm membrane and their fertilizing ability is maintained with proper supplementation of antioxidants [114]. Interestingly, a study has documented that proper administration enhances the antioxidant defense capability of the organism which is associated with modulation of the quality of the male ejaculate [115]. Selenoproteins such as selenophosphate synthase (SPS-2) and mitochondrial capsule selenoprotein (MCSeP) have been identified in testis [116]. The OS increases during pathological conditions and leads to lipid peroxidation which is negatively linked to the fertility potential of spermatozoa [106,117]. In addition, the high concentration of polyunsaturated fatty acids (PUFAs) also enhances lipid peroxidation which causes the sperm plasma membrane fluidity and integrity, thus affect the number of spermatozoa and their motility, which is necessary for sperm-oocyte fusion ability [106,117,118,119,120]. The OS also caused damage to sperm DNA, which is the main factor that contributes to the transmission of defective paternal DNA to a fetus [121]. The ROS produced malondialdehyde (MDA) from its action on membrane lipids which are mutagenic aldehydic lipid peroxidation products in seminal plasma and can be used as an infertility measurement tool [122,123]. The effect of Se on male sperm functions has been summarized in Figure 2.

#### 3.1.3. Role of Selenium in Ketosis and Fatty Liver Control

As discussed earlier, negative energy balance and oxidative stress cause many metabolic diseases, including ketosis in periparturient dairy cattle (Figure 1). It is well documented that periparturient cattle utilize their body fat to cope with the negative balance of energy [124]. The fatty acids are considered an essential source of energy in perinatal cattle. Thus, an increase in the concentration of ketone bodies and non-esterified fatty acids in plasma and decrease in blood glucose after delivery can be observed in dairy cattle. These changes may lead to oxidative stress and metabolic diseases like ketosis and fatty liver in dairy cattle [125,126]. The decrease in selenium concentration and total antioxidant capacity (TAOC) has been documented in cattle with clinical and subclinical ketosis [127]. Ren et al. has demonstrated that selenium improves glyconeogenesis and enhance the antioxidant system resulting in the reduction in the incidences of metabolic diseases such as fatty liver and kestosis in periparturient dairy cattle [128]. Furthermore, they documented that Se treatment down-regulated the expression of alpha S1 casein (CSN1S1), apolipoprotein A-I (APOA1), apolipoprotein C-II (APOC2) and up-regulated the macrophage-stimulating protein (MST1), chromogranin-A (CGA) in periparturient cattle. It has been documented that down-regulation of APOA1, APOC2, and CNS1S1 is associated with reducing the lipid activity, thus controlling the excessive fat mobilization, thereby reducing the chances of ketosis and fatty liver [129]. Moreover, the oxidized low-density lipoprotein (LDL) oxidation has been inhibited by MST1 which is essential for liver lipid and glucose metabolism [130].

## 4. Antioxidant Properties of Vitamin E and Their Role in Dairy Cattle Health

Vitamin E, a fat-soluble vitamin, is a strong antioxidant agent protecting cell membranes from the lipid peroxidation chain reaction [131] by acting in synergy with Se [132]. The cell membrane of immune cells consists of polyunsaturated fatty acids, which are sensitive to lipid peroxidation by ROS [112]. Exposure to aluminum created oxidative stress in mice, while antioxidative status was restored in mice followed by vitamin E injection [133]. Vitamin E readily exchanges and equilibrates between lipoproteins [134,135], and being an integral component of lipid membranes, it plays a protective role of lipid membranes from the attack of reactive oxygen [136,137]. It is the chain-breaking antioxidant and the first line of defense against lipid peroxidation, shielding cell membranes from free radical damage [138]. Vitamin E increases the functional efficiency of neutrophils to protect against oxidative damage following the intracellular killing of ingested bacteria [139]. α-Tocopherol, the most active form of vitamin E has been reported in many antioxidative processes [68,140,141]. Because of antioxidative property, vitamin E has an impact on the prevention of chronic diseases [142].

The α-Tocopherol is the most available bioactive form of vitamin E and has shown strong antioxidative and immunoregulatory properties in dairy cattle [143]. In addition, α-tocopherol prevents the proinflammatory status, enhances immunity and is linked to greater energy and reduces the susceptibility to infections in calves [144]. Similarly, Kuhn et al. documented that α-tocopherol (10 μ*M*) significantly inhibited the loss of bovine mammary endothelial cell barrier integrity induced by pro-oxidant. Furthermore, they reported that α-tocopherol has antioxidant properties which effectively prevents the bovine mammary endothelial cell damage and loss of function caused by oxidant challenge [145]. In addition, Mokhber-Dezfouli et al. reported that intramuscular injection of vitamin E significantly decreased the concentration of malondialdehyde (MDA), and lipid peroxidation and increased plasma antioxidant activity 4 hr after birth in calves [146]. The increased level of MDA and decreased level of total antioxidant capacity (TAOC) has been observed around parturition, which shows that the cows were under oxidative stress during the transition period [147]. Vitamin E improves the killing ability of neutrophils [148] and humoral immunity in calves [29], while its deficiency impairs the function of macrophages and neutrophils [149]. It has been documented that Vitamin E supplementation significantly improved the overall performance, energy metabolism and alteration in fat depot mass by reducing the oxidative stress in perinatal dairy cows [150]. For ease, the antioxidant properties of vitamin E are summarized in Table 2.

### Vitamin E Role in Cattle Health

A severe decrease in vitamin E level of blood during the transition period in dairy cattle has been observed [157,158]. Consequently, it has been investigated that the low level of vitamin E decreases in plasma during the periparturient period which is associated with intramammary infections [159]. In addition, the dairy cattle having vitamin E concentration in plasma lowers than 3mg/mL at calving were more susceptible to clinical mastitis [160]. The supplementation of vitamin E has been documented for its positive effect on antioxidant status; enhance immunity, overall peripartum reproductive performance and energy improvement of transition dairy cows [161]. Thus, vitamin E has gotten a growing interest, especially in preventing mammary infections of perinatal dairy cows [162]. Consistently, previously published studies had reported that vitamin E supplementation during perinatal period could reduce the chances of bovine mastitis [163,164]. Moreover, the administration of vitamin E in combination with Se enhanced the immunity and antioxidant status and showed more effective outcomes in preventing intramammary infections [160,165]. They further documented that somatic cell counts were significantly reduced in milk in response to vitamin E and Se treatment, which shows their effectiveness in reducing the incidences of mastitis. Consistently a study has documented that parenteral injections of vitamin E (2100 mg) for two weeks before and on calving day decrease the incidences of mastitis in dairy cows [166]. Similarly, a study documented that supplementation of α-Tocopherol (1 g/cow/day) for 30 days before and up to 60 days after calving significantly increased milk production and reduced the incidence of mastitis in Jersey cattle in India [167]. Vitamin E regulates the immunity and balances the oxidative status which is the main reason that exposes dairy cattle to udder infection and retained placenta [168,169,170]. Allison and Laven [171] documented that vitamin E is more effective against environmental udder pathogens such as *Escherichia coli* and *Streptococcus uberis* which are not normal inhabitants of skin or udder but gain entry during the periparturient period when the teat canals are open in dairy cows. Moreover, vitamin E reduces the oxidative stress in the udder and enhances immunity which usually declines during the transition period in dairy cattle [171].

In addition to antioxidant and immune regulatory properties, vitamin E also has an essential role in energy metabolism which is also a critical factor that exposes dairy cattle to oxidative stress and consequently to infections [172,173]. There is growing evidence that oxidative stress during perinatal period in dairy cows causes metabolism disturbance which may lead to the retained placenta [145]. Oxidative stress and immune disturbances are also considered the common factors that predispose periparturient cows to the retained placenta and consequent fertility issues [174,175,176]. The incidence of retained placenta was reported to be greater in dairy cattle having deficiency of vitamin E and Se [177]. A deficiency of vitamin E during the transition period relatively reduces the intake of green fodder, which induces the accumulation of lipid peroxides in the placenta, resulting in retained placenta [150,178]. It has been demonstrated that supplementation of a sufficient quantity of vitamin E in plasma during the periparturient period significantly reduced the cases of retained placenta [179]. Consistently, a study documented that vitamin E injections for seven days significantly reduced the occurrence of retained placenta [178]. Similarly, vitamin E injections (2100 mg) for two weeks before and on the day of calving prevent the incidences of retained placenta [154]. Consistently, another study documented that the injection of vitamin E (3000 IU) on the 21st day and the 5th day before parturition may contribute to reducing the incidence of retained placenta in dairy cattle [156]. Another study has shown that presupplementation of vitamin E decreased the incidence of stillbirth and retained placenta and improved the reproductive performance in dairy cattle by reducing oxidative stress [151,179].

## 5. Conclusions

To sum up, redox balance has an essential role in the regulation of several biological processes. However, when the imbalance occurs between the production of oxidants and the animal body natural antioxidant system, it may lead to serious health issues in periparturient dairy cattle. Therefore, the external antioxidant source may contribute to balance the situation of oxidative stress. Vitamin E and Se are well studied for their antioxidant and immune regulating properties. Thus the proper supplementation of Se and vitamin E during the periparturient period could be a good choice to relieve the oxidative stress and the consequent consequences related to health in dairy cattle.

## Figures and Tables

**Table 1:** Various types of Selenoproteins and their antioxidant properties.

Selenoproteins	Properties
Glutathione peroxidase 1	Cellular reduction of H_2_O_2_ [67,68].
Glutathione peroxidase 2	Reduction of peroxide in the gut [69].
Glutathione peroxidase 3	Reduction of peroxide in the blood [70].
Glutathione peroxidase 4	Causes the Reduction of hydrogen peroxide radicals and facilitates lipid peroxides to water and lipid alcohols and the cellular ferroptosis induced by iron [71].
Selenoprotein H	Responsible for Nuclear localization, which is associated with redox sensing and transcription [72,73].
Selenoprotein O	Mitochondrial protein consisted of a cytosine-nucleotide-nucleotide-uridine motif suggestive of the redox role [62].
Selenoprotein T	Deficiency leads to early embryonic lethality [74].
Selenoprotein W	Have a role of putative antioxidant which is important for muscle growth [75].
Selenophosphate synthetase 2	Selenophosphate synthetase 2 has an essential role in the biogenesis of all selenoproteins together with itself [76].

**Table 2:** The antioxidant properties of vitamin E.

Vitamin E Treatment	Possible Outcomes	References
Vitamin E parenteral administration	Prevented suppression of TAS and GPx	[19]
	Increased humoral immune response,	
	Enhanced daily growth in calves	
1 mg/kg of Vitamin E subcutaneous supplement	Enhance immunity and antioxidant system	[22]
	Regulated tumor necrosis factor-alpha (TNF-α), interleukin-1 (IL-1), interferon gamma (IFNγ), SOD and GPx in calves	
γ-tocopherol	Prevented cellular damage and loss of function of primary bovine mammary endothelial cells (BMECs) caused by oxidant challenge	[145]
	Decreased cell cytotoxicity and enhanced cell viability	
	Reduced lipid peroxidation and apoptosis caused oxidative challenge	
Vitamin E intramuscular injection (40 IU/kg body weight)	Enhanced antioxidant activity	[146]
	Suppressed lipid peroxidation	
	Decreased MDA values in plasma	
	Increased α-tocopherol in plasma of calves	
Vitamin E supplementation	Prevented oxidative stress caused by aluminum in rats	[133]
	Enhanced antioxidative status in rats	
	Decreased lipid peroxidation	
	Suppressed MDA concentration in plasma of rats	
	Decreased Plasma thiobarbituric acid-reacting substances (TBARS)	
Vitamin E supplementation	Enhanced antioxidative status and suppressed oxidative stress in perinatal cattle Enhanced GSH-Px concentration Decreased the SOD level	[43]
Vitamin E supplementation	Decreased the SOD, MDA and catalase (CAT) level Enhanced the activity of TAOC, phagocytic activity (PA) of granulocytes and lymphocyte proliferation assay (LPA) in transition dairy cows	[151]
Vitamin E supplementation (A review)	Enhanced the antioxidant capacity and immunity in transition dairy cattle	[112]
Vitamin E supplementation	Reduced tissue peroxidation in chicken	[152]
Vitamin E supplementation	Reduced lipid peroxidation in meat and enhance antioxidative status	[153]
Vitamin E supplementation	Enhanced the antioxidative status in dairy cattle	[154]
α-tocopherol supplementation	Enhanced antioxidant status Suppressed lipid peroxidation	[155]
Vitamin E injection	Decreased Plasma thiobarbituric acid-reacting substances (TBARS) in muscle	[156]
